# Supplementary material for: Stability of gut microbiome after COVID-19 vaccination in healthy and immuno-compromised individuals
Source: Life Sci Alliance. 2024 Feb 5;7(4):e202302529. doi: 10.26508/lsa.202302529 (PMC10844540; doi:10.26508/lsa.202302529)
Supplement: Supplementary file 1 [file LSA-2023-02529_TableS1.docx]

***Supplementary Table 1.*** *Paired sample alpha diversity analysis from different patient cohorts (healthy controls, HC, immune checkpoint therapy treated cancer patients, ICP, and primary immunodeficient patients PID). Statistical testing using paired Wilcoxon test, with bonferonni adjustment for multiple testing.*

| Diversity_Measure | Vaccine | Cohort | Timepoint_1 | Timepoint_2 | Samples | P_value | padj |
| --- | --- | --- | --- | --- | --- | --- | --- |
| chao1 | 1 | ICP | Pre-Dose | Acute | 8 | 0.10732756 | 0.32198267 |
| chao1 | 1 | ICP | Pre-Dose | Late | 8 | 0.83363488 | 1 |
| chao1 | 1 | ICP | Acute | Late | 12 | 0.84451927 | 1 |
| chao1 | 1 | PID | Pre-Dose | Acute | 3 | 1 | 1 |
| chao1 | 1 | PID | Pre-Dose | Late | 3 | 0.18144921 | 0.54434762 |
| chao1 | 1 | PID | Acute | Late | 5 | 0.78740649 | 1 |
| diversity_shannon | 1 | ICP | Pre-Dose | Acute | 8 | 0.36272651 | 1 |
| diversity_shannon | 1 | ICP | Pre-Dose | Late | 8 | 0.14148212 | 0.42444636 |
| diversity_shannon | 1 | ICP | Acute | Late | 12 | 0.22401538 | 0.67204615 |
| diversity_shannon | 1 | PID | Pre-Dose | Acute | 3 | 0.78926803 | 1 |
| diversity_shannon | 1 | PID | Pre-Dose | Late | 3 | 0.18144921 | 0.54434762 |
| diversity_shannon | 1 | PID | Acute | Late | 5 | 0.58963855 | 1 |
| chao1 | 2 | HC | Acute | Late | 3 | 0.78926803 | 0.78926803 |
| chao1 | 2 | ICP | Acute | Late | 28 | 0.67416589 | 0.67416589 |
| chao1 | 2 | PID | Acute | Late | 7 | 0.93264664 | 0.93264664 |
| diversity_shannon | 2 | HC | Acute | Late | 3 | 0.42267807 | 0.42267807 |
| diversity_shannon | 2 | ICP | Acute | Late | 28 | 0.06347195 | 0.06347195 |
| diversity_shannon | 2 | PID | Acute | Late | 7 | 0.55411313 | 0.55411313 |
| chao1 | 3 | HC | Pre-Dose | Acute | 9 | 0.63558612 | 1 |
| chao1 | 3 | HC | Pre-Dose | Late | 9 | 0.34282884 | 1 |
| chao1 | 3 | HC | Acute | Late | 11 | 0.44980379 | 1 |
| chao1 | 3 | ICP | Pre-Dose | Acute | 20 | 0.15589543 | 0.46768628 |
| chao1 | 3 | ICP | Pre-Dose | Late | 19 | 0.60124967 | 1 |
| chao1 | 3 | ICP | Acute | Late | 21 | 0.875701 | 1 |
| diversity_shannon | 3 | HC | Pre-Dose | Acute | 9 | 0.09720109 | 0.29160326 |
| diversity_shannon | 3 | HC | Pre-Dose | Late | 9 | 0.05802402 | 0.17407206 |
| diversity_shannon | 3 | HC | Acute | Late | 11 | 0.50487987 | 1 |
| diversity_shannon | 3 | ICP | Pre-Dose | Acute | 20 | 0.64074432 | 1 |
| diversity_shannon | 3 | ICP | Pre-Dose | Late | 19 | 0.73230683 | 1 |
| diversity_shannon | 3 | ICP | Acute | Late | 21 | 0.75441829 | 1 |
