# Supplementary material for: Stability of gut microbiome after COVID-19 vaccination in healthy and immuno-compromised individuals
Source: Life Sci Alliance. 2024 Feb 5;7(4):e202302529. doi: 10.26508/lsa.202302529 (PMC10844540; doi:10.26508/lsa.202302529)
Supplement: Supplementary file 2 [file LSA-2023-02529_TableS2.docx]

***Supplementary Table 2.*** *PERMANOVA analysis describing the influence of study variables on the variance seen in the composition of the gut microbiome samples from our patients.*

|  | Df | SumOfSqs | R2 | F | Pr(>F) |
| --- | --- | --- | --- | --- | --- |
| Patient | 58 | 5.25969001 | 0.68846997 | 4.59750338 | 0.001 |
| Vaccine | 2 | 0.03484019 | 0.00456042 | 0.88316197 | 0.644 |
| Timepoint | 2 | 0.02028177 | 0.00265479 | 0.51412148 | 0.903 |
| Patient:Vaccine | 43 | 0.8590415 | 0.1124447 | 1.01282775 | 0.603 |
| Patient:Timepoint | 92 | 1.03439397 | 0.13539756 | 0.57001733 | 0.891 |
| Vaccine:Timepoint | 3 | 0.05985228 | 0.0078344 | 1.0114614 | 0.568 |
| Patient:Vaccine:Timepoint | 36 | 0.33213056 | 0.04347441 | 0.4677311 | 0.937 |
| Residual | 2 | 0.03944937 | 0.00516375 | NA | NA |
| Total | 238 | 7.63967965 | 1 | NA | NA |
