# Supplementary material for: Stability of gut microbiome after COVID-19 vaccination in healthy and immuno-compromised individuals
Source: Life Sci Alliance. 2024 Feb 5;7(4):e202302529. doi: 10.26508/lsa.202302529 (PMC10844540; doi:10.26508/lsa.202302529)
Supplement: Supplementary file 3 [file LSA-2023-02529_TableS3.docx]

***Supplementary Table 3.*** *Paired sample analysis of the relative abundance of phylum from different patient cohorts (healthy controls, HC, immune checkpoint therapy treated cancer patients, ICP, and primary immunodeficient patients PID). Statistical testing using paired Wilcoxon test, with bonferonni adjustment for multiple testing; log2 fold-change (log2FC). Only those values with padj < 0.05 are shown.*

| Vaccine | Cohort | Timepoint_1 | Timepoint_2 | Phylum | foldchange | comparisons | P_value | padj | log2FC |
| --- | --- | --- | --- | --- | --- | --- | --- | --- | --- |
| 2 | ICP | Acute | Late | p__Firmicutes | 1.0257 | 28 | 1.77E-05 | 0.0003 | 0.0366 |
| 2 | ICP | Acute | Late | p__Verrucomicrobia | 1.9317 | 28 | 0.0006 | 0.0083 | 0.9499 |
| 3 | ICP | Acute | Late | p__Firmicutes | 1.0245 | 21 | 0.0002 | 0.0090 | 0.0349 |
| 2 | ICP | Acute | Late | p__Bacteroidetes | 0.8378 | 28 | 0.0007 | 0.0099 | -0.2553 |
| 3 | ICP | Acute | Late | p__Bacteria phylum incertae sedis | 1.4380 | 21 | 0.0004 | 0.0173 | 0.5241 |
| 3 | ICP | Pre-Dose | Late | p__Bacteria phylum incertae sedis | 1.6036 | 19 | 0.0006 | 0.0263 | 0.6814 |
| 2 | ICP | Acute | Late | p__Lentisphaerae | 2.2301 | 28 | 0.0025 | 0.0379 | 1.1571 |
| 3 | ICP | Acute | Late | p__Verrucomicrobia | 0.5737 | 21 | 0.0014 | 0.0615 | -0.8016 |
| 3 | ICP | Pre-Dose | Late | p__Verrucomicrobia | 0.4032 | 19 | 0.0017 | 0.0763 | -1.3103 |
| 1 | ICP | Acute | Late | p__Firmicutes | 0.9025 | 12 | 0.0025 | 0.1010 | -0.1480 |
| 1 | ICP | Acute | Late | p__Bacteroidetes | 1.5116 | 12 | 0.0025 | 0.1010 | 0.5960 |
| 2 | ICP | Acute | Late | p__Actinobacteria | 0.8219 | 28 | 0.0135 | 0.2023 | -0.2830 |
| 3 | HC | Pre-Dose | Acute | p__Bacteria phylum incertae sedis | 1.2644 | 9 | 0.0092 | 0.3386 | 0.3385 |
| 3 | ICP | Acute | Late | p__Proteobacteria | 1.1334 | 21 | 0.0091 | 0.4112 | 0.1806 |
| 1 | ICP | Pre-Dose | Acute | p__Bacteroidetes | 0.6999 | 8 | 0.0143 | 0.5706 | -0.5147 |
| 1 | ICP | Pre-Dose | Acute | p__Bacteria phylum incertae sedis | 0.5119 | 8 | 0.0143 | 0.5706 | -0.9660 |
| 1 | ICP | Pre-Dose | Late | p__Bacteria phylum incertae sedis | 0.4825 | 8 | 0.0143 | 0.5706 | -1.0513 |
| 2 | ICP | Acute | Late | p__Fusobacteria | 0.3655 | 28 | 0.0423 | 0.6347 | -1.4519 |
| 3 | ICP | Pre-Dose | Acute | p__Proteobacteria | 0.9265 | 20 | 0.0145 | 0.6513 | -0.1102 |
| 3 | HC | Pre-Dose | Acute | p__Proteobacteria | 1.4896 | 9 | 0.0178 | 0.6592 | 0.5750 |
| 3 | HC | Pre-Dose | Late | p__Proteobacteria | 1.2203 | 9 | 0.0178 | 0.6592 | 0.2873 |
| 1 | ICP | Pre-Dose | Acute | p__Proteobacteria | 0.7044 | 8 | 0.0209 | 0.8345 | -0.5056 |
| 1 | ICP | Pre-Dose | Late | p__Firmicutes | 0.9589 | 8 | 0.0209 | 0.8345 | -0.0606 |
| 1 | ICP | Pre-Dose | Late | p__Proteobacteria | 0.7439 | 8 | 0.0209 | 0.8345 | -0.4269 |
| 3 | HC | Pre-Dose | Late | p__Firmicutes | 0.9747 | 9 | 0.0244 | 0.9024 | -0.0370 |
| 3 | ICP | Pre-Dose | Acute | p__Fusobacteria | 1.7308 | 20 | 0.0209 | 0.9388 | 0.7914 |
